# Effect of SiO_2_ Content on the Enrichment of Perovskite in Ti-Bearing Blast Furnace Slag

**DOI:** 10.3390/ma19122613

**Published:** 2026-06-17

**Authors:** Lina Liu, Jiacheng Ding, Jun Fang, Lei Liu, Jinrui Zhang

**Affiliations:** 1 School of Emergency Management and Safety Engineering, North China University of Science and Technology, Tangshan 063210, China; llnty@ncst.edu.cn (L.L.); 17360812274@163.com (J.F.); 2 School of Mining Engineering, North China University of Science and Technology, Tangshan 063210, China15195037256@163.com (J.Z.)

**Keywords:** titanium-bearing blast furnace slag, process mineralogy, crystallization behavior, minerals, granularity, occurrence state, migration law

## Abstract

**Highlights:**

- Guided by process mineralogy theory, the effect of SiO_2_ content on the process grain size of perovskite was quantitatively investigated via polarized light microscopy.
- The occurrence mode, elemental association and migration behavior of titanium were further characterized using electron probe microanalysis (EPMA).

**Abstract:**

Titanium-bearing blast furnace slag is rich in high-melting-point titanium-containing minerals including perovskite, melilite and spinel, which result in the loss of titanium resources and hinder the comprehensive utilization of such slag. On this basis, combined with process mineralogy theories, this study adopted multiple characterization methods, including a polarized light microscope with transmitted and reflected light, XRD and EPMA. These simulations reveal that the bulk SiO_2_ content dictates titanium distribution among the mineral phases, thereby laying a solid foundation for the subsequent experiments. Meanwhile, quantitative analyses were performed on the microstructure, mineral composition and perovskite grain size of the slag. The occurrence state and migration law of titanium in the slag were systematically investigated. The results show that the microstructure of titanium-bearing blast furnace slag presents a porphyritic structure at different SiO_2_ levels. Its main mineral phases include perovskite, pyroxene, spinel and glass. Titanium is predominantly hosted in perovskite, with small amounts distributed in the pyroxene, spinel and glass phases. Reducing the SiO_2_ content facilitates the formation and grain coarsening of perovskite and promotes the migration of titanium from pyroxene and glass into perovskite. When the SiO_2_ content is 20%, the perovskite content reaches 44.3%. Among them, the proportion of grains larger than 40 μm is 59.94%, and the distribution ratio of titanium in perovskite is 86.78%. Under the experimental conditions of this study, 20% SiO_2_ is the optimal level. These findings can provide a theoretical reference for the efficient separation and recovery of titanium from titanium-bearing blast furnace slag.

## 1. Introduction

Titanium is an essential strategic metal characterized by low density, high strength, excellent corrosion resistance and superior high-temperature performance. Owing to these outstanding properties, it has been widely applied in aerospace [1,2,3], new energy systems [4,5,6], medical materials [7,8,9] and other high-end fields. Vanadium–titanium magnetite boasts global reserves of over 24 billion tons, serving as a crucial strategic mineral resource for titanium and vanadium. China holds more than 10 billion tons of such reserves, ranking first worldwide, and this ore dominates the occurrence of titanium resources in China [10]. During the smelting process of vanadium–titanium magnetite, most titanium elements enter the blast furnace slag and further combine with Ca, Mg, Si and other elements to form high-melting-point mineral phases. Titanium is not only dispersed in multiple mineral phases, but the generated titanium-bearing minerals also exhibit fine grain sizes. These characteristics greatly restrict the separation and enrichment of titanium components. Therefore, the efficient recovery of titanium resources remains difficult, which severely limits the large-scale comprehensive utilization of titanium-bearing blast furnace slag. At present, the cumulative stockpile of titanium-bearing blast furnace slag at Pangang Group has exceeded 80 million tons and keeps increasing year by year. The massive accumulation of slag not only causes environmental pollution and land occupation but also induces potential geological hazards such as landslides. Hence, it is urgent to realize the resource recycling of titanium-bearing blast furnace slag [11,12,13].

Multiple technical routes have been developed for titanium extraction from titanium-bearing blast furnace slag, mainly including hydrometallurgy [14,15], metal thermal reduction [16,17], carbon reduction method [18,19] and selective enrichment [20,21,22,23]. Traditional hydrometallurgy consumes large amounts of acid–base reagents, causing environmental pollution and severe equipment corrosion. Thermal reduction demands high temperatures and energy, and the obtained metals contain complex impurities that are hard to remove. Carbon reduction also suffers from severe pollution and high energy consumption. In comparison, the technical route of “selective enrichment–selective growth–selective separation” proposed by Sui et al. [24,25] exhibits superior application feasibility. The core of this technical route lies in the targeted enrichment of titanium. The main enriched mineral phases include perovskite, titanite and rutile. Among them, perovskite features a high titanium content and a stable crystal structure, resulting in excellent separation performance and making it the most valuable titanium-bearing phase for efficient recovery [26,27].

To this end, numerous studies have focused on the enrichment behavior of perovskite in titanium-bearing blast furnace slag, with an emphasis on chemical composition regulation and process parameter optimization. As the main constituent of perovskite, CaO acts as a pivotal controlling component in relevant research. Reasonable adjustment of slag alkalinity can change the ratio of CaO to SiO_2_, reconstruct the slag phase equilibrium system, and further facilitate the directional enrichment of titanium within the perovskite phase. Hu Mengjun et al. [28], Zhao Jian et al. [29] and Fu Nianxin et al. [30] verified that the perovskite content in titanium-bearing blast furnace slag increases significantly with rising alkalinity (CaO/SiO_2_). Yang Shuangping et al. [31] found that alkalinity regulation can markedly promote the crystallization of perovskite, which confirms that the crystallization characteristics can be optimized via compositional design. Li Yuhai et al. [32] further demonstrated that CaO exerts a dual effect on perovskite crystallization. Excessive CaO will increase slag viscosity and hinder crystal growth, whereas a proper amount of MnO can reduce slag viscosity and promote the precipitation and growth of perovskite crystals. Zhang Wenbo [33] revealed that increasing alkalinity (CaO/SiO_2_) effectively facilitates perovskite crystallization. In contrast, higher Al_2_O_3_ contents accelerate spinel phase formation and thus suppress the precipitation of perovskite. By combining thermodynamic calculation, multi-scale phase and microstructure characterization and in situ SHTT tests, Wu Zhu et al. [34] systematically confirmed that the CaO/SiO_2_ ratio is the core factor governing the crystallization performance of titanium-bearing blast furnace slag. They further clarified the dominant phase transformations and crystallization characteristics under different alkalinity levels.

The separation and recovery efficiency of perovskite are governed by its mineralogical parameters, including content, particle size and microstructure. However, existing studies on titanium-bearing blast furnace slag with varying alkalinity have primarily focused on qualitative analyses of perovskite content and particle size. There is limited systematic investigation on the quantitative characterization of perovskite content, particle size, morphology, titanium speciation, elemental correlation and migration behavior. To address this research gap, this study selected titanium-bearing blast furnace slag from Pangang Group as the research object. Combined with high-temperature smelting experiments and systematic process mineralogy analysis, XRD, polarized light microscopy and electron probe microanalysis were adopted to quantitatively characterize the mineral composition, particle size distribution, microscopic morphology and migration behavior of perovskite under different SiO_2_ contents. This work aims to reveal the intrinsic mechanism through which SiO_2_ regulates the precipitation and growth of perovskite and to determine the optimal control range of SiO_2_. Based on the present investigation, this paper supplements existing research by providing a quantitative analysis of perovskite content and process particle size, as well as the occurrence state and migration law of titanium in titanium-bearing blast furnace slag. It provides theoretical support for the efficient recovery and comprehensive resource utilization of titanium resources from such slag.

## 2. Materials and Methods

### 2.1. Experimental Materials

In this experiment, titanium-bearing blast furnace slag from Panzhihua Iron and Steel Group Co., Ltd., Panzhihua, China was used as the base slag for chemical composition analysis. The test procedures are described as follows: samples were collected from different positions of the blast furnace slag, pulverized and finely ground to a particle size of −0.074 mm using a vibrating grinding mill, and then sampled after sieving the pulverized specimens. Titration and spectrophotometry were adopted to test the chemical composition of the sieved samples to guarantee the representativeness of the determined chemical composition data of the slag. Table 1 lists the measured chemical compositions. Apart from TiO_2_, the main chemical components of the slag include CaO, SiO_2_, Al_2_O_3_ and MgO. The alkalinity, calculated as *w* (CaO)/*w* (SiO_2_), is 1.0, and the mass fraction of TiO_2_ is 19.3%, which is classified as medium-titanium titanium-bearing blast furnace slag.

Based on field data from Pangang, blast furnace slags with varying SiO_2_ contents were prepared using chemically pure reagents of CaO, SiO_2_, Fe_2_O_3_, TiO_2_, MgO and Al_2_O_3_. The SiO_2_ mass fractions were set at 17%, 20%, 23%, 26%, 29% and 32%, with the specific compositional details shown in Table 2.

### 2.2. Material Preparation

First, place the analytical-grade reagents in a drying oven, set the temperature to 120 °C and dry for 2 h to remove moisture. Mix the dried chemical components according to the proportions shown in Table 2, then place the prepared raw materials in a mixer and blend for 1 h. Abox-type atmosphere furnace (Model: KJ-A1700-8LZ, Honglu Technology Co., Ltd., Zhengzhou, Henan, China) was used to conduct remelting tests on the mixed samples. The furnace temperature was raised from room temperature to 1100 °C (heating time: 220 min), then further to 1500 °C (heating time: 200 min), and held at 1500 °C for 30 min to ensure complete melting of the samples. The temperature was subsequently lowered to 1100 °C and held for a further 30 min. Argon gas was introduced throughout the melting process, after which the samples were removed and allowed to cool naturally.

Each set of test samples was divided into two parts: one part was ground to −200 mesh and analyzed for the mineral composition of titanium-bearing blast furnace slag using an X-ray diffractometer (Model: Empyrean03030502, Malvern Panalytical Ltd., Malvern, Worcestershire, UK); the other part was prepared into thin sections using a cutting machine, a sectioning machine and a polishing machine. The mineral content, particle size distribution and morphological characteristics of titanium-bearing blast furnace slag were quantitatively determined using a German Zeiss research-grade polarizing microscope (Model: Scope.A1, Carl Zeiss AG, Oberkochen, Baden-Württemberg, Germany). Based on polarizing microscope analysis, a instrument electron probe microanalyzer (Model: JXA-823003040703, JEOL Ltd., Akishima, Tokyo, Japan) was used to test the chemical composition of each mineral and explore the occurrence state and migration patterns of titanium.

### 2.3. Research Methods for Thermodynamic Analysis of Titanium-Bearing Blast Furnace Slag

The crystallization thermodynamics of titanium-bearing blast furnace slag were simulated using the Equilib module in FactSage 8.0. FactSage, jointly developed by CRCT and GTT-Technologies, is an integrated thermochemical calculation system that operates on the principle of Gibbs free energy minimization and has been widely applied in thermodynamic simulations of metallurgical slag systems. Decterov et al. [35] established thermodynamic models for multi-component metallurgical slags via the Equilib module, while Kirschen et al. [36] simulated the phase equilibrium of the CaO-–SiO_2_–MgO–Al_2_O_3_–Cr_2_O_3_ system using the same module. In this study, the Equilib module was adopted to simulate the multi-component and multiphase equilibrium of titanium-bearing blast furnace slags with varying SiO_2_ contents at temperatures ranging from 1100 °C to 1500 °C, with the aim of elucidating the effect of SiO_2_ content on the crystallization process.

### 2.4. Quantitative Determination of Mineral Content in Titanium-Bearing Blast Furnace Slag

The mineral composition and content of titanium-bearing blast furnace slag are key factors governing titanium enrichment, process design, and extraction efficiency during titanium recovery. Polarizing microscopic analysis is the conventional technique for mineral quantitative characterization. Common quantitative methods under polarizing microscopy include the area method [37], point-counting method [38], and linear intercept method [39]. Mineral content was determined via the linear intercept method on a polarizing microscope (Model: Axioskop A1, Carl Zeiss AG, Oberkochen, Baden-Württemberg, Germany). This method arranges a set of parallel test lines on the thin section. The length ratio of line segments intercepted by individual mineral phases corresponds to their volume fraction, based on which the mineral content can be quantitatively calculated.

### 2.5. Quantitative Determination of Perovskite Process Particle Size of Titanium-Bearing Blast Furnace Slag

Process grain size refers to the actual geometric dimensions of mineral grains observed under a microscope [40]. It directly reflects the degree of mineral crystallization, grain growth trends and grindability, and serves as a key indicator for evaluating mineral coarsening, enrichment efficiency and recovery potential [41]. Under a polarizing microscope, methods for determining the process grain size of minerals include diameter measurement, area measurement and chord length measurement. The method employed in this experiment is chord length measurement. In this method, a chord interval is first defined, followed by the arrangement of a series of uniform parallel straight lines on the thin section; the chord lengths of mineral particles intercepted by these lines are recorded as the measured data. In this experiment, seven sets of chord intervals were defined, with a step size of 10 µm.

### 2.6. Qualitative Mineral Identification Method

The air-cooled slag samples were first finely ground to pass through a 74 μm sieve (−0.074 mm) and then dry-sieved. The mineral phases of the titanium-bearing blast furnace slag were qualitatively analyzed using an Empyrean X-ray diffractometer (Model No. 03030502). XRD measurements were performed under the following conditions: Cu Kα radiation with a Ni filter; solid-state detector; tube voltage of 40 kV; tube current of 40 mA; 2θ scanning range of 5–90°; scanning speed of 10°/min; incident X-ray wavelength of 0.15418 nm (λ = Cu Kα_1_).

### 2.7. Determination Method of Element Contents in Minerals

The air-cooled samples were fabricated as polished thin sections and coated with carbon. An electron probe microanalyzer (EPMA, Model JXA-8230, No. 03040703, JEOL Ltd., Tokyo, Japan) was used for in situ microarea elemental analysis of individual mineral grains. Prior to testing, the instrument was calibrated using six standard oxide specimens: TiO_2_, CaO, SiO_2_, MgO, Al_2_O_3_ and Fe_2_O_3_. Operating parameters: an acceleration voltage of 15 kV, a beam current of 2 × 10^−8^ A, and a beam diameter of 5 μm.

**Figure 1:**
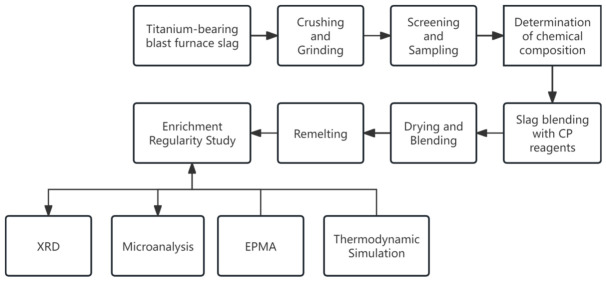
Schematic diagram of the experimental procedure.

A schematic diagram of the complete experimental procedure is shown in Figure 1.

## 3. Results and Discussion

### 3.1. Thermodynamic Analysis of Precipitation Characteristics of Titanium-Bearing Blast Furnace Slag Under Various SiO_2_ Contents

**Figure 2:**
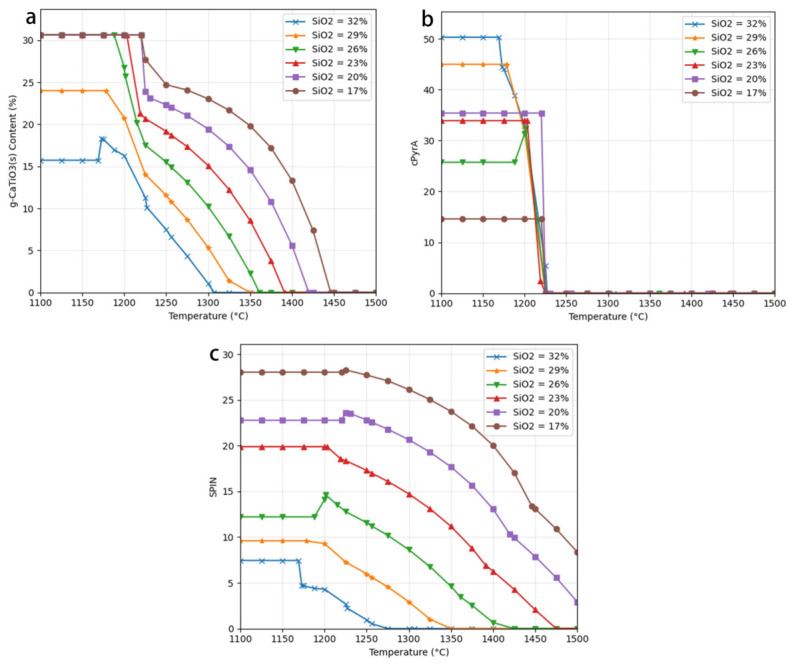
Perovskite precipitation law in slag system with different SiO_2_ contents: (**a**) perovskite; (**b**) pyroxene; (**c**) spinel.

To investigate the intrinsic influence of SiO_2_ content on the enrichment behavior of titanium-bearing blast furnace slag, thermodynamic simulations were performed on the crystallization process in titanium-bearing blast furnace slag under different SiO_2_ content conditions. The results are shown in Figure 2.

Figure 2 shows that both the onset and completion temperatures of perovskite crystallization gradually increase with decreasing SiO_2_ content. The crystallization onset temperatures are 1300 °C < 1325 °C < 1350 °C < 1375 °C < 1400 °C < 1425 °C, and the crystallization end temperatures are 1169 °C < 1179 °C < 1188 °C < 1203 °C < 1220 °C = 1220 °C. When the SiO_2_ content is reduced to 20%, the end temperature (1220 °C) remains unchanged. The yield of perovskite increases significantly, reaching a steady state at a SiO_2_ content of 26%. The SiO_2_ content has little effect on the onset temperature of pyroxene crystallization, which remains around 1220 °C; however, as the SiO_2_ content decreases, the end temperature of pyroxene crystallization increases from 1169 °C to 1220 °C, and the overall crystallization content of pyroxene shows a decreasing trend. As the SiO_2_ content decreases, both the onset and completion temperatures of spinel crystallization increase, and the crystallization yield of spinel rises.

### 3.2. Quantitative Analysis of Titanium-Bearing Blast Furnace Slag Minerals

**Figure 3:**
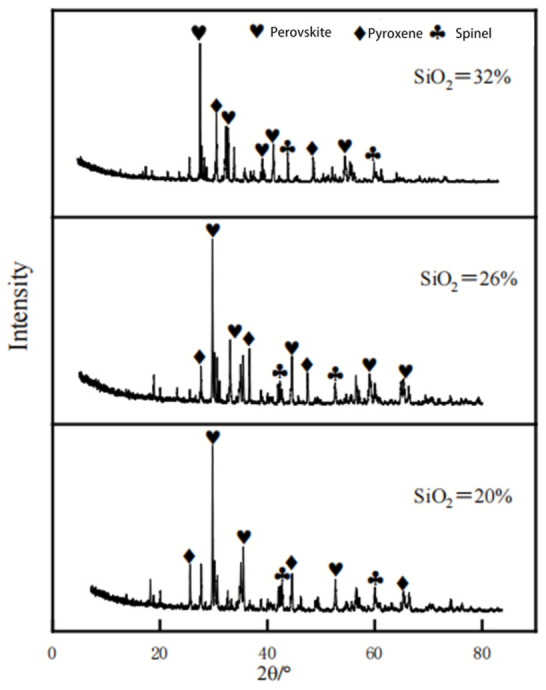
XRD patterns of titanium-bearing blast furnace slag with different SiO_2_ contents.

X-ray diffraction (XRD) was employed to analyze the phase composition of titanium-bearing blast furnace slag under different SiO_2_ content conditions. The raw XRD data were processed using MDI Jade 9 software, including background subtraction, peak fitting and multiple iterations of optimization to minimize measurement errors. Phase identification and characterization were carried out using the S/M peak method. The XRD patterns of titanium-bearing blast furnace slag under different SiO_2_ content conditions are shown in Figure 3.

Figure 3 shows the XRD patterns of titanium-bearing blast furnace slag with different SiO_2_ contents. No new diffraction peaks appeare, nor is the disappearance of existing diffraction peaks observed. This indicates that variations in SiO_2_ contents do not alter the primary mineral types within the slag system. The main phases in titanium-bearing blast furnace slag are perovskite, pyroxene and a small amount of spinel, indicating that the mineral composition of the slag exhibits good stability within this range.

**Figure 4:**
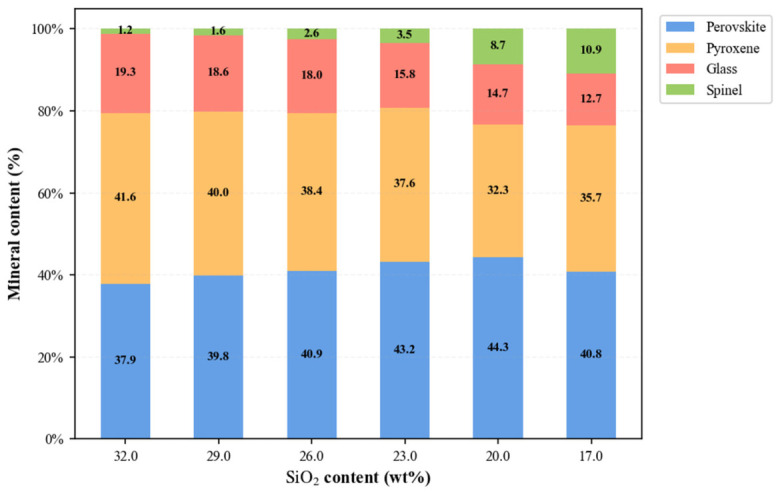
Mineral content of titanium-bearing blast furnace slag with different SiO_2_ contents.

Building on the qualitative XRD analysis of titanium-bearing blast furnace slag, a quantitative mineralogical analysis of slag samples with r different SiO_2_ contents was conducted using a polarizing microscope. The intercept lengths of individual minerals were measured using the straight-line method, and their mass fractions were calculated based on the mineral densities. The densities selected were 4.0 g·cm^−3^ for perovskite, 3.4 g·cm^−3^ for pyroxene, 2.5 g·cm^−3^ for glass and 3.6 g·cm^−3^ for spinel. Mineral content diagrams were plotted based on the statistical results of the mineral content in titanium-bearing blast furnace slag under different SiO_2_ contents, as shown in Figure 4.

Figure 4 shows that the perovskite content generally increases with decreasing SiO_2_ content in the slag. When the SiO_2_ content is 32%, crystallization thermodynamic simulations indicate that the initial crystallization temperature of perovskite is 1300 °C. At high temperatures, higher SiO_2_ content leads to increased slag viscosity, making it difficult for Ca^2+^ and TiO_3_^2−^ to diffuse and migrate, resulting in a lower yield of perovskite. As the SiO_2_ content decreases, slag viscosity decreases relatively, accelerating the diffusion of Ca^2+^ and TiO_3_^2−^, which promotes the nucleation and growth of perovskite crystals, leading to an increase in perovskite content. When the SiO_2_ content is 20%, the perovskite content reaches its maximum value.

As the SiO_2_ content decreases, the overall content of pyroxene tends to decrease. When the SiO_2_ content is 32%, thermodynamic crystallization simulations indicate that the initial crystallization temperature of pyroxene is lower than that of perovskite. As the temperature decreases, slag viscosity decreases. Under high SiO_2_ conditions, the formation of silicate tetrahedra is promoted, and these tetrahedra combine with elements such as Fe, Mg and Ca to form pyroxene. As the SiO_2_ content decreases, on the one hand, slag viscosity decreases, allowing more Ca^2+^ to combine with TiO_3_^2−^ to form perovskite; on the other hand, under low-SiO_2_ conditions, the formation of silicate tetrahedra is reduced, leading to an overall decrease in pyroxene content.

The overall proportion of spinel in the slag remains relatively low. Its content exhibits only a slight gradual increase with rising SiO_2_ levels, peaking at 10.90% when the SiO_2_ content reaches 17%. Spinel primarily crystallizes from the reaction between MgO and Al_2_O_3_; consequently, variations in SiO_2_ content within the system have a negligible effect on spinel formation. As the SiO_2_ content decreases, the proportion of Al_2_O_3_ in the slag increases correspondingly. This indicates that changes in spinel content are predominantly governed by the increasing Al_2_O_3_ content, rather than by the regulation of SiO_2_ components.

The glassy content decreases obviously with the reduction of SiO_2_ content. As the SiO_2_ content decreases from 32% to 17%, the glass content declines from 19.3% to 12.7%. High SiO_2_ content corresponds to high slag viscosity, which weakens the migration of various elements and promotes the generation of an amorphous glassy phase. With the continuous reduction of SiO_2_ content, slag viscosity gradually decreases. This facilitates the crystallization of perovskite and pyroxene, thereby further reducing the glass content.

### 3.3. Effect of SiO_2_ Content on the Microstructure of Titanium-Bearing Blast Furnace Slag

Using a polarizing microscope, we analyzed the microstructural features, mineral morphology, and particle sizes of six groups of titanium-bearing blast furnace slag with different SiO_2_ contents.

**Figure 5:**
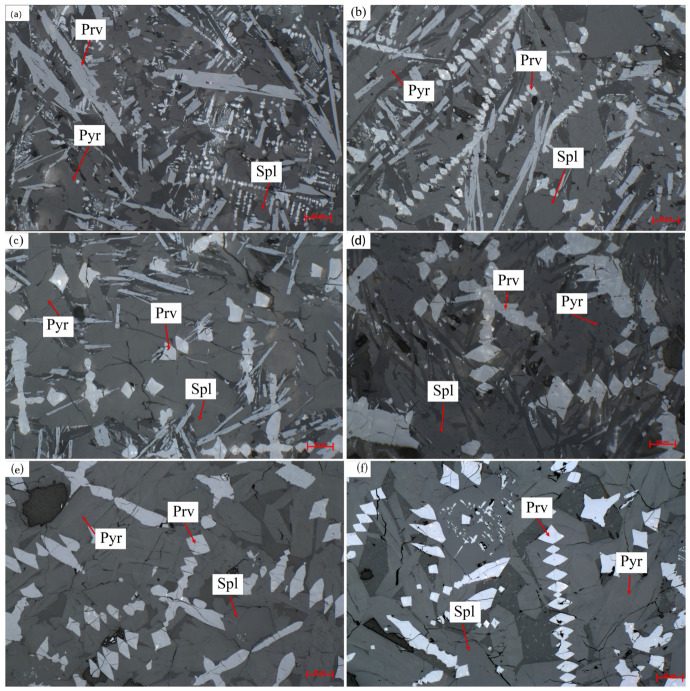
Microstructure of titanium-bearing blast furnace slag with different SiO_2_: (**a**) SiO_2_ 32%; (**b**) SiO_2_ 29%; (**c**) SiO_2_ 26%;(**d**) SiO_2_ 23%;(**e**) SiO_2_ 20%; (**f**) SiO_2_ 17%.

Figure 5 shows that titanium-bearing blast furnace slag exhibits a porphyritic structure under different SiO_2_ content conditions. The slag consists of a glassy matrix as well as phenocrysts of perovskite, pyroxene and spinel, and a small number of pores can be observed in all samples.

When the SiO_2_ content is 32%, the perovskite particle size is extremely uneven. It is mainly composed of fine grains that form dendritic aggregates with subhedral and cruciform morphologies, while a small number of coarse grains exhibit elongated shapes. Cruciform perovskite is difficult to dissociate into single particles during crushing and grinding, which exerts a certain influence on the subsequent separation of perovskite. Pyroxene exhibits a columnar morphology, with particle sizes ranging from 20 µm to 40 µm and a uniform distribution. Spinel exists as idiomorphic and semi-automorphic crystals, with particle sizes between 20 µm and 30 µm, and is mostly distributed along the slag boundaries. When the SiO_2_ content is 29%, perovskite shows an uneven grain size distribution. Fine cruciform grains coarsen into fine rhomboidal grains, while long prismatic grains show no obvious variation. Pyroxene maintains a stable morphology with a slight decrease in content. Spinel grain size increases to 30–50 µm with a non-uniform distribution. When the SiO_2_ content decreases to 26%, the cumulative proportions of perovskite grains larger than 30, 40 and 60 μm are 39.03%, 15.32% and 0.81%, respectively. These results indicate that the fine perovskite particles are mainly coarsened to a size range of 30–40 μm. When the SiO_2_ content is 20%, the perovskite grain size increases significantly. Rhomboidal grains are further coarsened, and some evolve into rod-like morphologies, with most grain sizes distributed in the range of 40–70 µm. Pyroxene grain size increases to 30–50 µm with a uniform distribution, whereas spinel shows no obvious changes. When the SiO_2_ content is 17%, the overall grain size of perovskite decreases, and some finer-grained perovskite appears. The finer grains are mainly nodular aggregates, with small perovskite grains ranging from 5 µm to 10 µm. The content of pyroxene increases, and pyroxene exhibits a columnar morphology with grain sizes of 40–50 µm. The grain size of spinel increases to 40–50 µm, and most crystals show an isometric shape.

These observations indicate that, as the SiO_2_ content decreases, the particle size of the perovskite first increases and then decreases, with the distribution becoming progressively more uniform. The morphology evolves from fine dendritic particles to coarser columnar and rhombohedral shapes, which facilitates the separation of the perovskite. The coarsening is most pronounced when the SiO_2_ content is 20%.

### 3.4. Effect of SiO_2_ Content on the Particle Size of Perovskite

**Figure 6:**
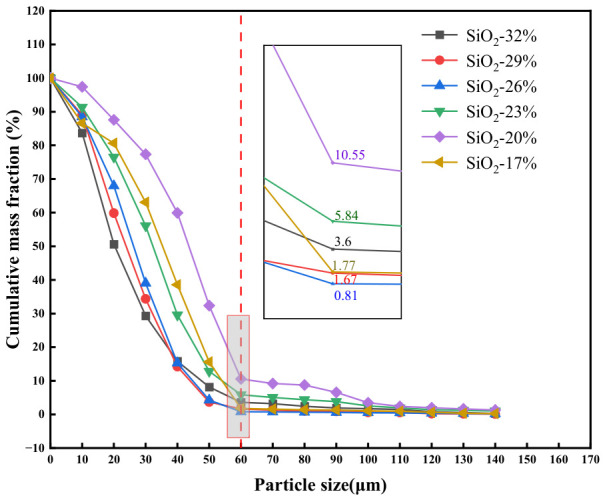
Process particle size curves of perovskite in titanium-bearing blast furnace slag under different SiO_2_ contents.

Table 3 shows the particle size and cumulative distribution of perovskite at different SiO_2_ levels. Based on the cumulative data, the particle size distribution curve was plotted, as shown in Figure 6.

Figure 6 shows that the SiO_2_ content has a significant effect on the particle size distribution of perovskite. As the SiO_2_ content gradually decreases from 32% to 17%, the perovskite particle size exhibit an overall trend of coarsening first and then refining. When the SiO_2_ content is 32%, the grain size of perovskite is mainly concentrated in the fine-grain range. The cumulative content of grains larger than 10 μm is 83.68%, the cumulative content at the 20 μm grain size reaches 50.63%, the cumulative content above 30 μm is 29.32%, that above 40 μm is only 15.84%, and that above 60 μm is merely 3.6%. Perovskite particles are mostly distributed below 30 μm. This is attributed to the high viscosity of SiO_2_ at high temperatures, which promotes the formation of a glass phase in the melt [42]. It further restricts the diffusion and migration of Ca^2+^ and TiO_3_^2−^, inhibits the nucleation and growth of perovskite crystals, and ultimately leads to the precipitation of perovskite in the form of fine and dispersed particles. As the SiO_2_ content decreases to 26%, the cumulative content of grains above 30 μm reaches 39.03%, the cumulative content above 40 μm is 15.32%, and the cumulative content above 60 μm is 0.81%. This indicates obvious coarsening of perovskite grains larger than 30 μm. When the SiO_2_ content further decreases to 20%, perovskite exhibits the most significant coarsening effect. The cumulative contents of grains above 40 μm and 60 μm increase to 59.94% and 10.55%, respectively. At low SiO_2_ content, the slag viscosity decreases, and the crystal growth process becomes dominant. The crystal growth rate is remarkably accelerated, resulting in prominent coarsening of perovskite. When the SiO_2_ content continues to decrease to 17%, the perovskite particle size shows a refinement trend, with a reduced proportion of coarse particles. The cumulative fractions of particles above 40 μm and 60 μm decrease to 38.56% and 1.77%, respectively.

### 3.5. Occurrence State of Titanium in Titanium-Bearing Blast Furnace Slag

**Figure 7:**
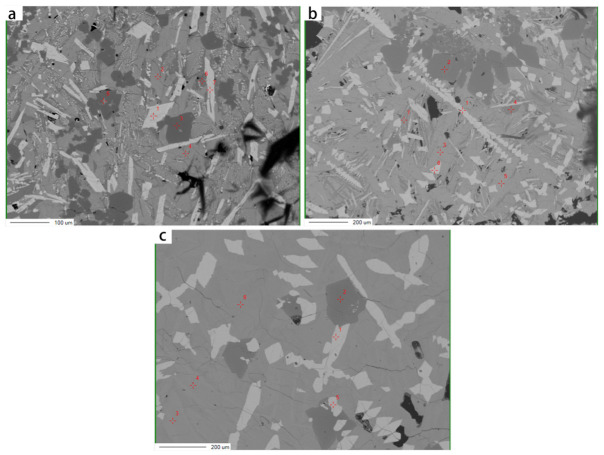
Electron probe microanalysis (EPMA) point analyses of titanium-bearing blast furnace slag with different SiO_2_ contents: (**a**) 32% SiO_2_; (**b**) 26% SiO_2_; (**c**) 20% SiO_2_.

**Figure 8:**
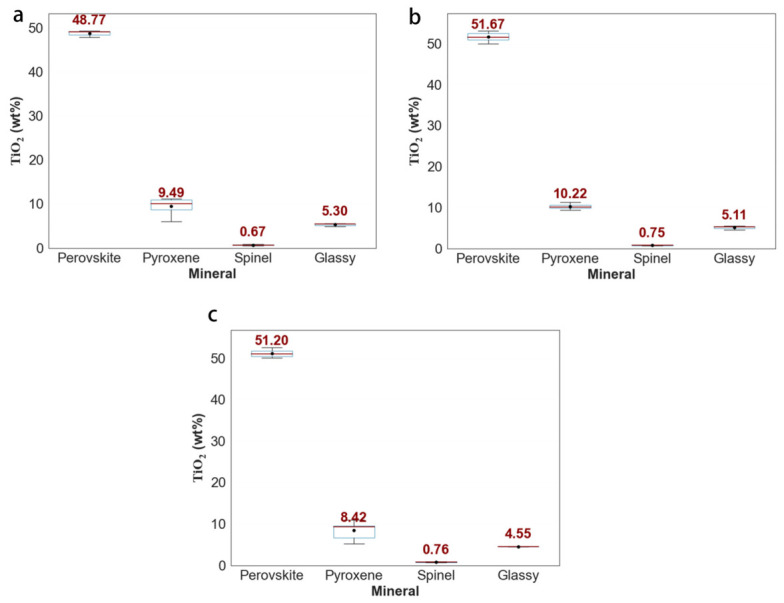
Distribution box plots of TiO_2_ under different SiO_2_ contents (**a**) SiO_2_ 32%; (**b**) SiO_2_ 26%; (**c**) SiO_2_ 20%.

Using an electron probe microanalyzer (EPMA), we performed quantitative analyses of the chemical compositions of minerals under different slag SiO_2_ content conditions and plotted the results as box plots, as shown in Figure 7 and Figure 8 and Table 4. 

Figure 8 shows that Ti is mainly distributed in perovskite, followed by pyroxene, with small amounts present in the glassy phase and spinel under different SiO_2_ contents. As the SiO_2_ content decreases, the average Ti content in perovskite increases by 2.43%, while that in pyroxene decreases by 1.07%; the Ti content in spinel and the glassy phase remains basically unchanged.

Based on the distribution characteristics of TiO_2_ in various mineral phases mentioned above, the element correlations in the minerals of titanium-bearing blast furnace slag were analyzed. The results are presented in Figure 8.

**Figure 9:**
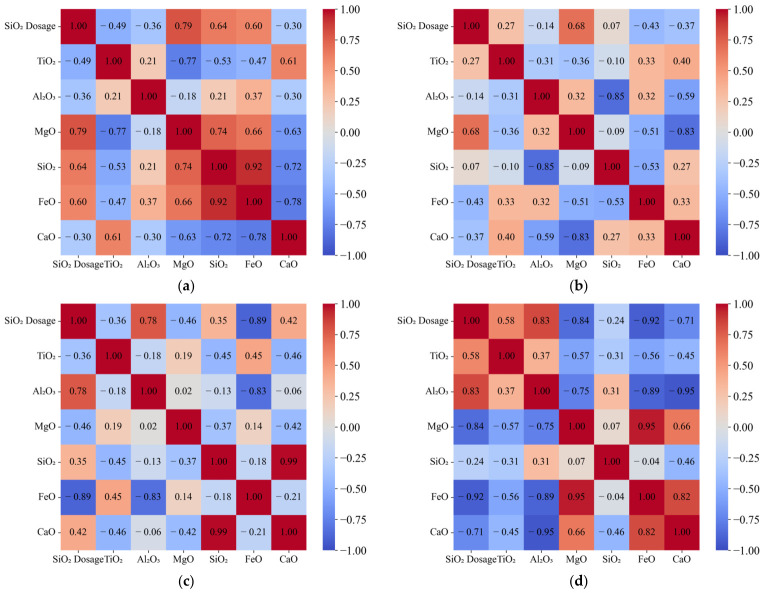
Element correlation analysis of minerals in titanium-bearing blast furnace slag: (**a**) perovskite; (**b**) pyroxene; (**c**) spinel; (**d**) glassy phase.

Figure 9 shows that Ti exists as an independent mineral in perovskite. The TiO_2_ content shows a negative correlation with the SiO_2_ content (R = −0.49), indicating that a decrease in SiO_2_ content promotes the enrichment of titanium in the perovskite phase. It exhibits a strong positive correlation with CaO, a strong negative correlation with MgO, and a weak correlation with other components. In pyroxene, the TiO_2_ content shows a weak positive correlation with SiO_2_ content (R = 0.21), indicating that a decrease in SiO_2_ content inhibits the migration of TiO_2_ into pyroxene. It exhibits a strong negative correlation with MgO and FeO. The chemical formula of pyroxene is Ca(Mg, Fe^2+^, Fe^3+^, Al)[(Si,Al)_2_O_6_]. Based on the diagonal rule of isomorphism and ion types, Ti primarily substitutes for Fe^3+^ through an isomorphic manner and does not readily substitute Mg. In the spinel phase, the TiO_2_ content is negatively correlated with the SiO_2_ content, weakly positively correlated with MgO, and weakly negatively correlated with Al. The general chemical formula of spinel is MgO·Al_2_O_3_. Based on the diagonal rule of isomorphism, Ti replaces Al^3+^ in spinel through isomorphic substitution. In the glassy phase, the TiO_2_ content is positively correlated with the SiO_2_ content.

### 3.6. Migration Law of Titanium Element in Titanium-Bearing Blast Furnace Slag

**Figure 10:**
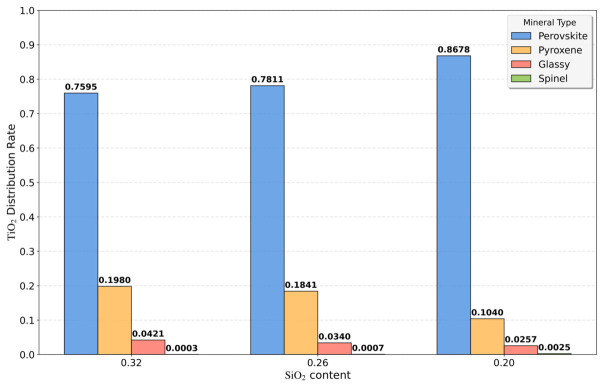
Migration characteristics of titanium in titanium-bearing blast furnace slag.

Based on the quantitative mineral analysis and electron probe microanalysis (EPMA) results of titanium-bearing blast furnace slag, the distribution characteristics of titanium in the slag were determined, as presented in Table 5 and Figure 10.

Analysis of Table 4 and Figure 10 indicates that with the gradual reduction of SiO_2_ content in the system, the distribution ratio of TiO_2_ in the perovskite phase of titanium-bearing blast furnace slag presents a distinct increasing trend. By comparison, the distribution ratios in the pyroxene, spinel and glassy phases decline to varying degrees. These variation tendencies demonstrate that reducing SiO_2_ content can effectively regulate the precipitation behavior of mineral phases and the elemental partitioning in molten slag. Such evolution facilitates the migration and enrichment of TiO_2_ from the pyroxene, spinel and glassy phases into the perovskite lattice via preferential solid solution, thereby promoting the directional enrichment of titanium within the perovskite phase.

## 4. Conclusions

This study systematically investigated the effects of SiO_2_ content on the mineral composition, particle size distribution, microstructure and the occurrence and migration characteristics of titanium in titanium-bearing blast furnace slag. The main conclusions are summarized as follows:

1. SiO_2_ content exerts a distinct influence on the crystallization temperatures of perovskite, pyroxene, and spinel in titanium-bearing blast furnace slag.
2. While SiO_2_ content does not change the overall structure or main phase types of titanium-bearing blast furnace slag, it exerts a notable influence on the crystallinity of individual mineral phases. Slags with varying SiO_2_ contents exhibit a porphyritic texture, with the main crystalline phases including perovskite, pyroxene, spinel and a glassy phase. As SiO_2_ content decreases, perovskite content first rises and then slightly decreases, reaching a peak of 44.30% at a SiO_2_ content of 20%. By contrast, the pyroxene content decreases continuously, the glassy phase content declines remarkably, and the spinel content displays a slowly rising trend. A high SiO_2_ content at elevated temperatures increases melt viscosity, which in turn restrains the precipitation of perovskite.
3. With the reduction of SiO_2_ content, the particle size of perovskite in titanium-bearing blast furnace slag first coarsens and then refines. The coarsening effect is the most significant at 20% SiO_2_, and the proportion of particles larger than 40 μm increases significantly. Meanwhile, the morphology of perovskite transforms from fine dendritic aggregates to coarse rhombic crystals, which is beneficial to the subsequent separation and extraction of titanium resources.
4. In titanium-bearing blast furnace slag, titanium is mainly hosted in perovskite, followed by pyroxene, spinel and glassy phase. The TiO_2_ content in perovskite is negatively correlated with SiO_2_ content, while the TiO_2_ content in pyroxene shows a weak positive correlation with SiO_2_. The reduction of SiO_2_ content promotes the migration and enrichment of titanium toward the perovskite phase.
5. Comprehensive consideration of perovskite content, particle size, crystal morphology and titanium migration behavior indicates that the optimal SiO_2_ mass fraction in this experimental system is 20%. This work provides a theoretical reference for the efficient extraction of titanium from titanium-bearing blast furnace slag.

## Figures and Tables

**Table 1:** Main components of titanium-bearing blast furnace slag from Pangang Group (wt%).

CaO	SiO_2_	TiO_2_	Al_2_O_3_	Fe_2_O_3_	MgO	Na_2_O	V_2_O_5_	MnO	Cr_2_O_3_
25.4	25.2	19.3	12.2	1.93	8.29	0.785	0.29	0.44	0.24

**Table 2:** Composition ratios of titanium-bearing blast furnace slag and setting of related parameters.

Sample	SiO_2_	Fe_2_O_3_	CaO	TiO_2_	MgO	Al_2_O_3_	Alkalinity	Hold. Temperature	Hold. Time
TR-1	32	8	24	18	10	8	0.75	1100	30
TR-2	29	8	24	18	10	11	0.83	1100	30
TR-3	26	8	24	18	10	14	0.92	1100	30
TR-4	23	8	24	18	10	17	1.0	1100	30
TR-5	20	8	24	18	10	20	1.2	1100	30
TR-6	17	8	24	18	10	23	1.4	1100	30

**Table 3:** Perovskite particle size distribution %.

	Grain Size	≤10	10~20	20~30	30~40	40~50	50~60	≥60	Distribution Type
SiO_2_									
32%		16.32	33.05	21.31	13.48	7.66	4.58	3.60	Range
		100	83.68	50.63	29.32	15.84	8.18	3.6	Cumulative
29%		10.98	29.17	25.47	20.16	10.54	2.01	1.67	Range
		100	89.02	59.85	34.38	14.22	3.68	1.67	Cumulative
26%		11.54	20.47	28.96	23.71	11.05	3.46	0.81	Range
		100	88.46	67.99	39.03	15.32	4.27	0.81	Cumulative
23%		8.67	14.81	20.43	26.52	16.72	7.01	5.84	Range
		100	91.33	76.52	56.09	29.57	12.85	5.84	Cumulative
20%		2.57	9.85	10.21	17.43	27.58	21.81	10.55	Range
		100	97.43	87.58	77.37	59.94	32.36	10.55	Cumulative
17%		5.74	13.61	17.54	24.55	22.95	13.84	1.77	Range
		100	94.26	80.65	63.11	38.56	15.61	1.77	Cumulative

**Table 4:** Average EPMA compositions of minerals as a function of bulk SiO_2_ content.

SiO_2_	Mineral	Average Element Content					
		TiO_2_	Al_2_O_3_	MgO	SiO_2_	FeO	CaO
32	Perovskite	48.77	0.41	0.68	0.42	7.33	37.40
	Pyroxene	11.56	14.72	11.07	32.68	4.53	25.35
	Spinel	0.67	63.51	27.66	0.37	5.60	0.26
	Glassy	5.30	31.12	0.22	43.45	0.70	21.26
26	Perovskite	51.67	0.35	0.07	0.00	0.45	45.59
	Pyroxene	12.97	14.99	8.05	30.47	5.30	28.61
	Spinel	0.75	62.50	29.32	0.04	6.19	0.01
	Glassy	5.11	24.41	0.48	45.72	0.94	24.61
20	Perovskite	51.20	0.56	0.05	0.00	0.67	42.20
	Pyroxene	8.42	16.32	8.75	33.40	5.68	27.27
	Spinel	0.76	60.59	29.04	0.06	9.32	0.03
	Glassy	4.55	12.60	2.46	44.18	1.72	33.04

**Table 5:** Element composition of minerals under different SiO_2_ contents.

SiO_2_	Mineral	Content	TiO_2_ Mass Fraction	TiO_2_ Load of Mineral	TiO_2_ Distribution Rate
32%	Perovskite	37.86	48.77	18.464	75.95%
	Pyroxene	41.64	11.56	4.814	19.80%
	Spinel	1.17	0.67	0.008	0.03%
	Glassy	19.33	5.30	1.024	4.21%
26%	Perovskite	40.93	51.67	21.149	78.11%
	Pyroxene	38.44	12.97	4.986	18.41%
	Spinel	2.60	0.75	0.020	0.07%
	Glassy	18.03	5.11	0.921	3.40%
20%	Perovskite	44.30	51.20	22.682	86.78%
	Pyroxene	32.27	8.42	2.717	10.40%
	Spinel	8.69	0.76	0.066	0.25%
	Glassy	14.74	4.55	0.671	2.57%

## Data Availability

The original contributions presented in this study are included in the article. Further inquiries can be directed to the corresponding author.
